# Magnitude of serum β-hydroxybutyrate elevations and myocardial glucose suppression in fluorine-18 fluorodeoxyglucose positron emission tomography for cardiac sarcoidosis

**DOI:** 10.1093/ehjimp/qyag161

**Published:** 2026-09-16

**Authors:** Lamis El Harake, Richard Weinberg, Ike Okwuosa, Ryan Avery, Susan Lehrer, Paul C Cremer

**Affiliations:** Division of Cardiology, Bluhm Cardiovascular Institute, Chicago, IL, USA; Division of Cardiology, Bluhm Cardiovascular Institute, Chicago, IL, USA; Bluhm Cardiovascular Institute, Feinberg School of Medicine, Northwestern University, 676 N St Clair St: Suite 700, Chicago, IL, USA; Division of Cardiology, Bluhm Cardiovascular Institute, Chicago, IL, USA; Bluhm Cardiovascular Institute, Feinberg School of Medicine, Northwestern University, 676 N St Clair St: Suite 700, Chicago, IL, USA; Bluhm Cardiovascular Institute, Feinberg School of Medicine, Northwestern University, 676 N St Clair St: Suite 700, Chicago, IL, USA; Department of Radiology, Feinberg School of Medicine, Northwestern University, Chicago, IL, USA; Division of Cardiology, Bluhm Cardiovascular Institute, Chicago, IL, USA; Division of Cardiology, Bluhm Cardiovascular Institute, Chicago, IL, USA; Bluhm Cardiovascular Institute, Feinberg School of Medicine, Northwestern University, 676 N St Clair St: Suite 700, Chicago, IL, USA

## Abstract

This retrospective cohort study evaluated the relationship between serum β-hydroxybutyrate (BHB) levels and myocardial glucose suppression in 74 patients undergoing fluorine-18 fluorodeoxyglucose positron emission tomography for suspected cardiac sarcoidosis. The average BHB concentration prior to FDG administration was 0.94 ± 0.78 mmol/L. The septal-to-blood pool uptake ratio (SUVmax_septal/SUVmean_blood pool) ranged from 0.50 to 0.88, with a median of 0.66 (Quartile 1 = 0.58; Quartile 3 = 0.73). No significant association was found between BHB levels and the septal-to-blood pool uptake ratio (*r* = 0.09; *P* = 0.48), suggesting a metabolic plateau regarding myocardial glucose utilization once adequate ketosis is achieved. These preliminary findings support a threshold effect rather than a dose-dependent relationship between serum BHB levels and myocardial glucose suppression following appropriate dietary preparation.

## Graphical Abstract

**Graphical Abstract:**
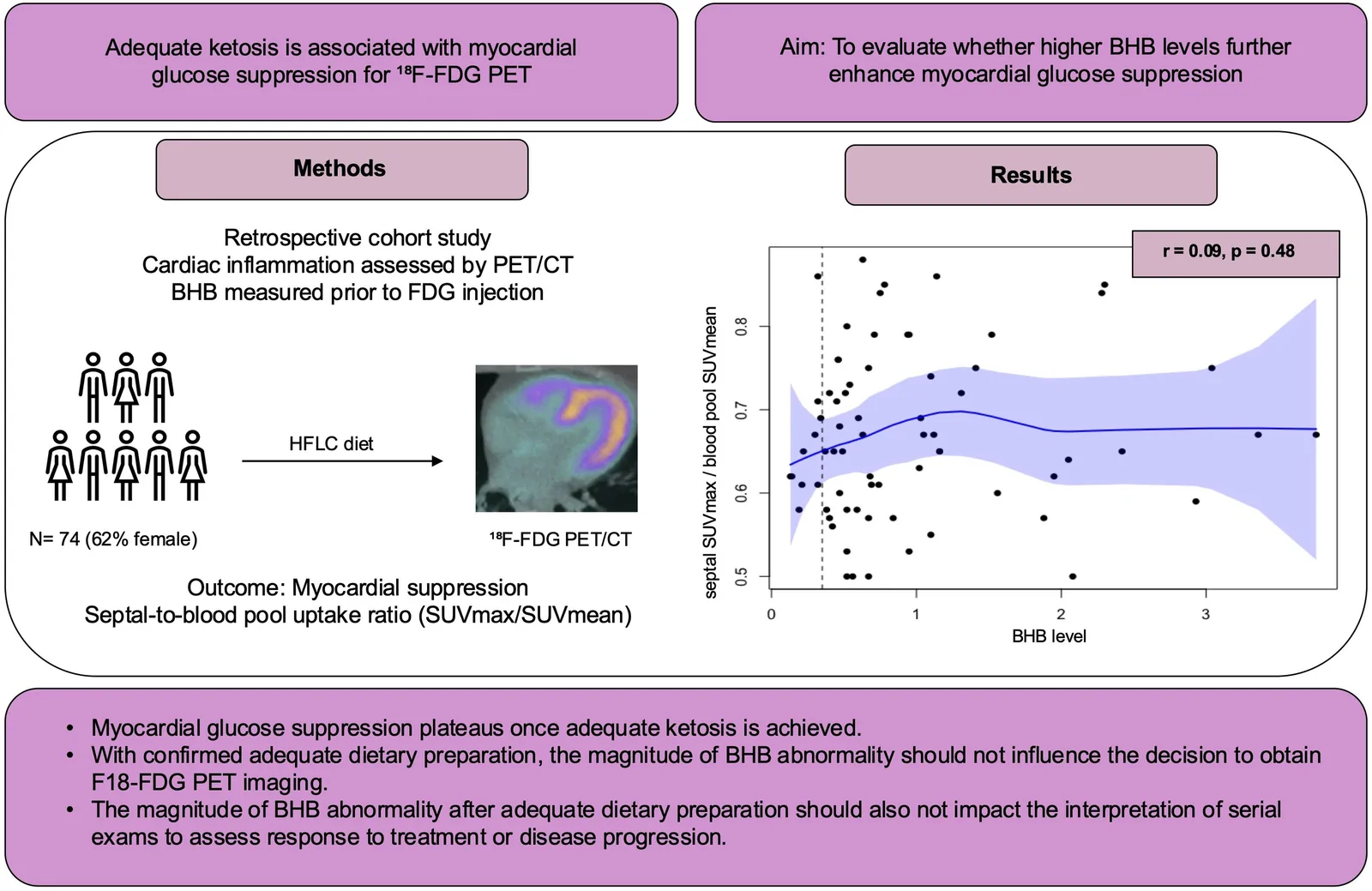

## New knowledge gain and clinical implications

- Routine β-hydroxybutyrate (BHB) measurement can help verify adequate patient preparation before FDG injection and prevent non-diagnostic scans.
- With adequate ketogenic dietary preparation, higher BHB concentrations do not further enhance myocardial glucose suppression, supporting a metabolic plateau rather than a dose-dependent relationship.
- For patients undergoing serial fluorine-18 fluorodeoxyglucose positron emission tomography scans, these preliminary results suggest that differences in BHB levels at the time of image acquisition should not impact comparative interpretation, provided that the dietary preparation has been appropriate.

## Introduction

Fluorine-18 fluorodeoxyglucose positron emission tomography (^18^FDG-PET) is used to evaluate cardiac inflammation due to its ability to detect metabolically active inflammatory cells via [^18^F]-FDG uptake, a glucose analogue.^1,2^ However, physiological glucose uptake by non-inflamed cardiomyocytes can obscure pathological uptake, making suppression of normal myocardial metabolism essential for accurate image interpretation. To suppress physiologic myocardial glucose uptake, patient preparation protocols include a low-carbohydrate, high-fat diet, prolonged fasting, and administration of unfractionated heparin to enhance lipolysis and elevate circulating free fatty acids.^3^ These interventions promote metabolic shifts towards fatty acid oxidation, reducing myocardial FDG uptake. Despite adherence to these protocols, incomplete myocardial suppression occurs in up to 20% of patients.^2^

β-Hydroxybutyrate (BHB), a ketone body produced during fatty acid metabolism, has emerged as a promising biomarker of effective myocardial suppression.^2,4^ Recent studies have shown a negative correlation between circulating BHB levels and myocardial [^18^F]-FDG uptake, with a proposed threshold of ≥0.35 mmol/L associated with a high specificity for adequate suppression.^2,4–5^ However, it is unclear whether the relationship between BHB levels and myocardial glucose suppression is linear or represents a threshold effect, which may be particularly important in patients undergoing serial ^18^FDG-PET exams to assess progression of disease or response to treatment. Therefore, the aim of the current study is to evaluate whether higher BHB levels are associated with greater degrees of myocardial glucose suppression.

## Methods

### Study population and patient preparation

Consecutive patients referred for cardiac ^18^F-FDG PET/CT at Northwestern Memorial Hospital between February 2024 and October 2025 for evaluation of suspected cardiac inflammation were included. All patients had BHB and fasting blood glucose levels measured prior to FDG injection as part of the standardized imaging protocol. A total of 74 patients met the inclusion criteria and were included in the final analysis. The study was approved by the Northwestern University Institutional Review Board (STU00222964) and conducted in accordance with institutional guidelines with a waiver of the requirement for informed consent.

### Patient preparation and biomarker collection

All patients were instructed to follow a high-fat, low-carbohydrate diet for all meals on the day prior to PET imaging, followed by a fasting period of at least 8 h after dinner, with only water permitted. If patients reported significant carbohydrate intake or non-compliance with the HFLC diet, the scan was rescheduled. Patients received a single intravenous bolus of unfractionated heparin at 50 IU/kg (maximum 5000 IU), administered 15 min prior to ^18^F-FDG injection. For patients already receiving systemic anticoagulation, a reduced dose of 12 IU/kg was used. Serum BHB was measured using a standardized laboratory assay.

### Image protocol

Imaging was performed using a PET/CT scanner (Biograph Vision 600, Siemens Healthineers, Erlangen, AG). FDG imaging was conducted 60–90 min following injection of 8–10 mCi of ^18^F-FDG (dose adjusted for body mass index). PET data were acquired over 15 min in list mode and reconstructed using 3D ordered subset expectation maximization per manufacturer protocol.

### Imaging analysis

Images were reviewed using Corridor4DM software (INVIA Medical Imaging Solutions) by two independent readers. On fused, CT attenuation-corrected FDG PET/CT images, a 1 cm diameter, 3D circular region of interest (ROI) was drawn in the centre of the left ventricle to measure mean blood pool standardized uptake value (SUVmean). Myocardial SUVmax was measured using circular 3D ROIs placed in the mid-septal and mid-lateral walls, avoiding areas of abnormal FDG uptake. Additional SUVmean measurements were taken from the ascending aorta (at the level of the pulmonary artery) and from the liver.

### Statistical analysis

Continuous variables were summarized as mean ± standard deviation (SD), and categorical variables were reported as counts and percentages. To evaluate the relationship between BHB levels and myocardial FDG uptake, an index was calculated by dividing septal myocardial SUVmax by blood pool SUVmean. This SUVmax/SUVmean ratio was used as a quantitative marker of myocardial suppression. The correlation between BHB levels and this suppression index was assessed using linear regression and Pearson’s correlation coefficient. Locally estimated scatterplot smoothing (LOESS) curves were also generated to visualize the relationship. All analyses and visualizations were performed using R version 4.4.3 (The R Foundation for Statistical Computing, Vienna, Austria).

## Results

### Patient characteristics

*Table 1* summarizes the baseline characteristics of the study population. A total of 74 patients were included in the analysis, of whom 46 (62.1%) were male with a mean age of 63.4 ± 10.9 years. Hypertension was present in 62.2% of patients, coronary artery disease (CAD) in 51.4%, and dyslipidaemia in 82.4%. Diabetes mellitus was reported in 29.7%, while 68.9% had a history of heart failure and 29.7% had atrial fibrillation. Twenty-nine patients (39.2%) were current or former smokers, and 18 (24.3%) reported a family history of CAD. All patients demonstrated adequate myocardial suppression, as determined by expert reader evaluation.

**Table 1:** Baseline characteristics of the study population (***n* = 74)**

Baseline characteristics of the study cohort	Total (*n* = 74)
Demographic variables	
Age (years)	63.36 (10.86)
Sex *(male)*	46 (62.1%)
Weight (kg)	87.58 (22.19)
Height (cm)	173.25 (8.99)
BMI	29.01 (6.60)
BSA	2.01 (0.26)
Cardiovascular risk factors	
Hypertension	46 (62.2%)
CAD	38 (51.4%)
Diabetes	22 (29.7%)
HbA1c (%)	6.06 (1.35)
Dyslipidaemia	61 (82.4%)
Ever smoked	29 (39.2%)
Family history of CAD	18 (24.3%)
Atrial fibrillation	22 (29.7%)
Heart failure	51 (68.9%)
Medications	
Anti-platelets	28 (37.8%)
ACEi/ARB	49 (66.2%)
Statins	54 (73.0%)
Beta-blockers	50 (67.6%)
Insulin	6 (8.1%)
Laboratory values	
Beta-hydroxybutyrate (mmol/L)	0.94 (0.78)
Glucose (mg/dL)	92.36 (19.23)

Summary of clinical characteristics, comorbidities, and metabolic parameters of patients referred for cardiac ^18^F-FDG PET/CT imaging. Continuous variables are presented as mean ± SD and categorical variables as counts and percentages.

### BHB level and myocardial uptake

Of the 74 included patients, 71 had analysable scans for quantitative assessment of myocardial uptake. Three patients demonstrated diffuse, heterogeneous myocardial FDG uptake, precluding accurate SUV measurements; all three were considered abnormal scans consistent with myocardial involvement and were not classified as non-diagnostic. Among the 74 scans, 53 (70.7%) showed no evidence of myocardial inflammation, and 22 (29.3%) were positive for focal cardiac inflammation.

The average BHB concentration prior to FDG administration was 0.94 ± 0.78 mmol/L. Prior studies have reported a negative correlation between BHB levels and myocardial [^18^F]-FDG uptake, with a proposed threshold of 0.35 mmol/L serving as a specific criterion of adequate myocardial suppression. In our cohort, 11 of 74 patients (14.9%) had BHB levels below 0.35 mmol/L. Among these patients, the septal-to-blood pool uptake ratio was 0.65 ± 0.08 compared with 0.69 ± 0.15 in those with BHB ≥ 0.35 mmol/L (*P* = 0.45). All 11 scans were considered diagnostic.

To assess whether higher BHB levels are associated with increased suppression, we analysed the correlation between BHB concentration and the septal-to-blood pool uptake ratio (SUVmax_septal/SUVmean_blood pool). Among our cohort, the uptake ratio ranged from 0.50 to 0.88, with a median of 0.66 (Q1 = 0.58; Q3 = 0.73). Pearson’s correlation analysis yielded an *r* value of 0.09 (*P* = 0.48), indicating no significant linear association. The LOESS curve generated visualizes this relationship, confirming the absence of a consistent trend across the range of BHB levels (*Figure 1*).

**Figure 1:**
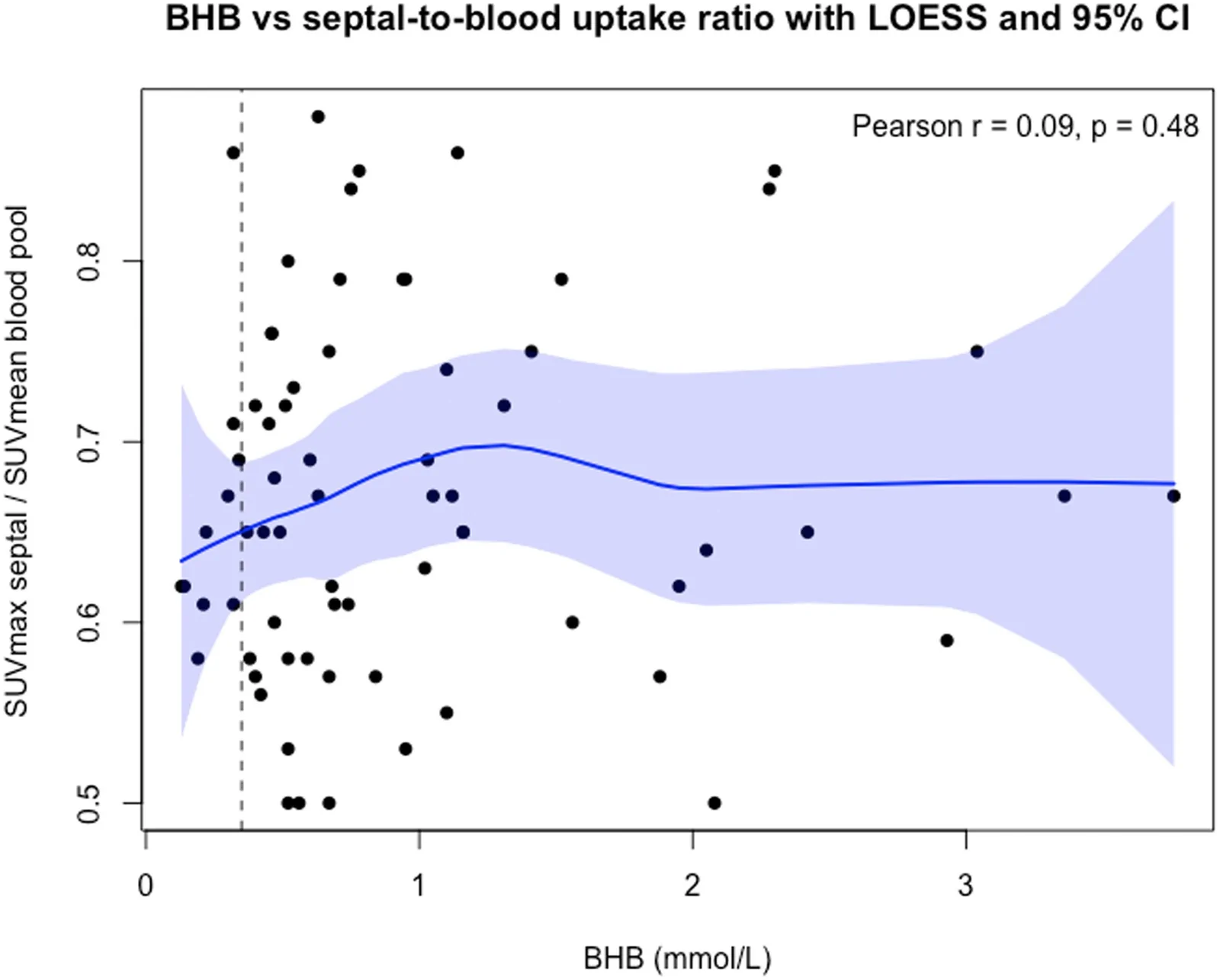
Relationship between serum BHB levels and septal-to-blood pool SUV ratio. LOESS curve illustrating the relationship between BHB concentration (mmol/L) and the septal myocardial SUVmax to blood pool SUVmean ratio (SUVmax septal/SUVmean LVBP). No significant linear association was observed (Pearson’s *r* = 0.09; *P* = 0.48), supporting a threshold effect rather than a dose-dependent relationship. The dashed vertical line denotes a BHB level of 0.35 mmol/L. Pearson correlation coefficient is shown in the upper right corner.

## Discussion

In this retrospective cohort study of patients referred for ^18^F-FDG PET/CT to evaluate for cardiac sarcoidosis, the aim was to assess whether the magnitude of BHB level elevation was correlated with myocardial glucose suppression. Of note, BHB levels were not associated with greater myocardial glucose suppression among patients who had abnormal BHB levels, even including patients who had levels lower than a previously proposed threshold. These findings support a threshold effect rather than a dose–response relationship, suggesting that once adequate ketosis is achieved, further increases in BHB may not further enhance myocardial glucose suppression. This observation is clinically important because it indicates that the assessment of response to treatment or progression of disease should not be impacted by the magnitude of ketosis induced with appropriate dietary preparation.

The ratio of septal SUVmax to blood pool SUVmean was used as a marker of suppression, in line with prior work, and demonstrated that suppressed myocardium consistently shows FDG uptake below background blood pool activity.^1^ Several studies have evaluated serum BHB as a predictor of adequate myocardial glucose suppression. A retrospective study by Madamanchi *et al.* reported that a serum BHB ≥ 0.35 mmol/L was associated with adequate suppression with 88% sensitivity and 61% specificity, and a cross-sectional study by Alfawara *et al.* found a similar cut-off (≥0.335 mmol/L) with 84.9% sensitivity and 92.6% specificity.^2,4^ In contrast to these studies, which used laboratory serum BHB values, Hartikainen *et al.* evaluated a point-of-care BHB testing device and found that BHB ≥ 0.35 mmol/L was associated with adequate myocardial glucose suppression with 90% specificity and 56% sensitivity, highlighting the potential feasibility of bedside testing workflows.^5^ Although prior studies suggest that BHB measured as early as 4 h before injection performs similarly to later timepoints, same-day, point-of-care testing may be more convenient for outpatient settings.^2^ This approach can help avoid unnecessary radiotracer injection and reduce the risk of non-diagnostic scans.

Our findings support incorporating BHB measurement into routine PET protocols as a pre-scan check for appropriate imaging. If a patient’s BHB is <0.35 mmol/L, the clinical team may consider reconfirming dietary adherence before proceeding with injection. However, if appropriate dietary adherence is confirmed, imaging should not be further delayed given that higher BHB levels do not confer additional benefit regarding myocardial glucose suppression.

Our study population included only patients undergoing FDG-PET for suspected cardiac sarcoidosis, which may limit generalizability to other patient groups or inflammatory indications. Additionally, our sample size was not sufficient to evaluate differences between diabetic and non-diabetic patients, and this distinction may influence clinical decision-making regarding BHB levels. Although BHB is a promising metabolic biomarker, its role within PET workflows still requires refinement. Future prospective, multicentre studies should determine whether patient-specific factors, preparation protocols, or disease types (e.g. sarcoidosis vs. endocarditis) require individualized BHB targets. The potential utility of other circulating ketones in assessing myocardial glucose suppression also warrants investigation.

In conclusion, BHB levels help inform whether the acquisition of myocardial FDG imaging is appropriate. With adequate dietary preparation, increasingly abnormal serum BHB levels do not further enhance myocardial glucose suppression. This insight has implications for patients undergoing serial ^18^F-FDG PET exams. Specifically, if the BHB level is abnormal, the magnitude of abnormality should not impact myocardial glucose suppression and comparison of scans. As a readily available metabolic biomarker, BHB adds value to the ^18^F-FDG PET workflow and may help improve image quality, reduce unnecessary imaging, and streamline care.

## Lead author biography


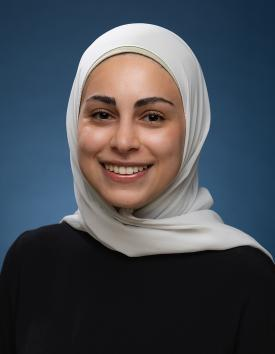


Lamis El Harake received her MD from the American University of Beirut in 2024 and is currently a postdoctoral research fellow in the Division of Cardiology at Northwestern University, where she has been working since December 2024. Her research focuses on multimodality cardiovascular imaging.

## Data Availability

The data underlying this article cannot be shared publicly due to the privacy of individuals that participated in the study. The data will be shared on reasonable request to the corresponding author.
